# Retraction notice for: “Long non-coding RNA-ROR aggravates myocardial ischemia/reperfusion injury” [Braz J Med Biol Res (2018) 51(6): e6555]

**DOI:** 10.1590/1414-431X2020e6555retraction

**Published:** 2021-06-02

Weiwei Zhang^1^, Ying Li^2^, Peng Wang^1^

^1^Department of Cardiology, Dezhou People’s Hospital, Dezhou, China

^2^Interventional Center, Dezhou People’s Hospital, Dezhou, China

**Retraction for:** Braz J Med Biol Res | doi: http://10.1590/1414-431X20186555 | PMID: 29694511 | PMCID: PMC5937723

The authors would like to retract the article “Long non-coding RNA-ROR aggravates myocardial ischemia/reperfusion injury” that was published in volume 51, no. 6 (2018) (Epub April 23, 2018) of the Brazilian Journal of Medical and Biological Research.

After the publication of this study, the corresponding author requested its retraction due to “the identification of data fabrication”. The Editors decided to retract this paper to avoid further damage to the scientific community.

The Brazilian Journal of Medical and Biological Research remains vigilant to prevent misconduct and reinforces the Journal’s commitment to good scientific practices. We regret the unprofessional behavior of the authors involved.

Correspondence: Weiwei Zhang: <zhangweiwei3832@126.com>

